# Lactate concentration gradient from right atrium to pulmonary artery

**DOI:** 10.1186/cc3741

**Published:** 2005-06-10

**Authors:** Guillermo Gutierrez, Lakhmir S Chawla, Michael G Seneff, Nevin M Katz, Hasan Zia

**Affiliations:** 1 Professor of Medicine and Anesthesiology, Pulmonary and Critical Care Medicine Division and Department of Medicine, The George Washington University Medical Center Washington, DC, USA; 2 Assistant Professor of Anesthesiology and Medicine, Critical Care Medicine Division, Department of Anesthesiology, The George Washington University Medical Center Washington, DC, USA; 3 Associate Professor Anesthesiology, Critical Care Medicine Division, Department of Anesthesiology, The George Washington University Medical Center Washington, DC, USA; 4 Clinical Professor of Surgery, Cardio-Thoracic Critical Care, Department of Surgery, The George Washington University Medical Center Washington, DC, USA; 5 Senior Resident, Division of General Surgery, Department of Surgery, The George Washington University Medical Center Washington, DC, USA

## Abstract

**Introduction:**

We compared simultaneous measurements of blood lactate concentration ([Lac]) in the right atrium (RA) and in the pulmonary artery (PA). Our aim was to determine if the mixing of right atrial with coronary venous blood, having substantially lower [Lac], results in detectable decreases in [Lac] from the RA to the PA.

**Methods:**

A prospective, sequential, observational study was conducted in a medical-surgical intensive care unit. We enrolled 45 critically ill adult individuals of either sex requiring pulmonary artery catheters (PACs) to guide fluid therapy. Immediately following the insertion of the PAC, one paired set of blood samples per patient was drawn in random order from the PAC's proximal and distal ports for measurement of hemoglobin concentration, O_2 _saturation (SO_2_) and [Lac]. We defined Δ[Lac] as ([Lac]_ra _- [Lac]_pa_), ΔSO_2 _as (S_ra_O_2 _- S_pa_O_2_) and the change in O_2 _consumption (ΔVO_2_) as the difference in systemic VO_2 _calculated using Fick's equation with either S_ra_O_2 _or S_pa_O_2 _in place of mixed venous SO_2_. Data were compared by paired Student's t-test, Spearman's correlation analysis and by the method of Bland and Altman.

**Results:**

We found S_ra_O_2 _> S_pa_O_2 _(74.2 ± 9.1 versus 69.0 ± 10.4%; p < 0.001) and [Lac]_ra _> [Lac]_pa _(3.9 ± 3.0 versus 3.7 ± 3.0 mmol.l^-1^; p < 0.001). Δ[Lac] correlated with ΔVO_2 _(r^2 ^= 0.34; p < 0.001).

**Conclusion:**

We found decreases in [Lac] from the RA to PA in this sample of critically ill individuals. We conclude that parallel decreases in SO_2 _and [Lac] from the RA to PA support the hypothesis that these gradients are produced by mixing RA with coronary venous blood of lower SO_2 _and [Lac]. The present study is a preliminary observation of this phenomenon and further work is needed to define the physiological and clinical significance of Δ[Lac].

## Introduction

Pulmonary artery (PA) blood comprises the mixed venous effluent from all organs, with the notable exception of the lungs. PA O_2 _saturation (S_pa_O_2_) has been promoted as an index of tissue oxygenation [1,2] because it is thought to be related to the average end capillary blood PO_2 _[3].

In a prior study [4], we measured the O_2 _saturation (SO_2_) of right atrial blood (S_ra_O_2_) and S_pa_O_2 _in samples drawn from the proximal and distal ports of PA catheters (PACs) placed in critically ill patients. We noted that S_pa_O_2 _was consistently lower than S_ra_O_2 _by approximately 5%. Others have noted a similar step-down in O_2 _saturation from the right atrium (RA) to the PA [5,6], and continuous measurements in critically ill patients have shown a similar difference between S_pa_O_2 _and central venous (CV) O_2 _saturation (S_cv_O_2_) of approximately 7% [7].

The RA to PA O_2 _saturation gradient (defined as ΔSO_2 _= S_ra_O_2 _- S_pa_O_2_) is likely the result of mixing atrial blood with highly desaturated blood entering the right heart chambers from the coronary veins. This includes blood flowing from the coronary sinus, the great cardiac vein and other major epicardial veins.

As a result of myocardial lactate extraction from the coronary circulation, the CV lactate concentration ([Lac]_cv_) is the lowest of any venous blood [8,9]. In the present study we compare blood lactate concentration ([Lac]) in paired samples drawn from the proximal and distal ports of PACs placed in critically ill patients ([Lac]_ra _and [Lac]_pa_) to establish whether we could also detect a decreasing lactate concentration gradient from right atrium to pulmonary artery (Δ[Lac] = [Lac]_ra _- [Lac]_pa_).

## Methods

This was a prospective, sequential study performed in the George Washington University Hospital intensive care unit. The George Washington University Institutional Review Board approved the study and informed consent was obtained from the patient or from the next of kin.

The data presented were culled from a subset of patients enrolled in a previous study [4]. We enrolled individuals older than 18 years of age of either sex in whom their physicians determined that a PAC was required to guide fluid therapy. Enrollment in the study occurred at the time the patient or the nearest relative consented to the introduction of the PAC. On the basis of their medical history, we excluded patients with uncorrected valvular incompetence, intra-cardiac shunting or those who required insertion of the pulmonary artery catheter through the femoral vein.

A 7.5 French, 5 lumen, 110 cm length, PAC with the right atrial lumen positioned 30 cm from the tip (Edwards Lifesciences, Irvine, CA, USA) was inserted through the internal jugular vein or the subclavian vein using a percutaneous sheath introducer (8.5 French; Edwards Lifesciences). The insertion technique is described elsewhere [4]. Care was taken to place the distal port catheter in the PA and the proximal port in the RA.

Immediately after the insertion of the PA catheter, each patient had one set of paired blood samples drawn in rapid succession, and in random order, from the proximal and distal port. We took proximal port blood to be representative of RA blood, whereas distal port blood was considered to be PA blood. The first 2 ml of blood drawn for each sample were discarded to prevent contamination with flushing fluid. Blood samples were drawn with the catheter balloon deflated to avoid contamination of the distal port sample with pulmonary capillary blood. Arterial O_2 _saturation was determined from a previously *in vivo *calibrated pulse oximeter.

Blood samples were placed on ice and taken to a central laboratory for measurement of [Lac] (Ektachem 950 IRC Chemistry Analyzer with a Vitros Products lactate slide, Ortho-Clinical Diagnostic, Inc., Rochester, NY, USA), hemoglobin concentration ([Hb]) and O_2 _saturation (ABL700 Radiometer America Inc., Westlake, OH, USA). We measured cardiac output (CO) by the thermodilution method as the average of three sequential determinations.

Systemic O_2 _delivery (DO_2_), O_2 _consumption (VO_2_), O_2 _extraction ratio (ERO_2_), double product (DP; heart rate (HR) × mean arterial pressure (MAP)) and left ventricular stroke work index (LVSWI) were computed using standard formulae. We defined ΔVO_2 _as the difference in systemic VO_2 _calculated with Fick's equation with either S_pa_O_2 _or S_ra_O_2 _in place of the mixed venous SO_2 _(S_v_O_2_); ΔVO_2 _= Q_pa _× 13.4 × [Hb] × (S_ra_O_2 _- S_pa_O_2_) ml.min^-1^.

Paired Student's t-test was used to compare atrial to PA measurements. [Lac]_ra _and [Lac]_pa _were compared by Spearman's correlation analysis [10]. The method of Bland and Altman [11] was used to investigate the effect of lactate concentration on the differences between paired observations. The relationships between Δ[Lac] and ΔSO_2_, ΔVO_2 _and other hemodynamic parameters were analyzed by Spearman's correlation analysis. Data are shown as mean ± SD with p < 0.05 denoting a significant difference.

## Results

We enrolled 45 patients in the study, including 18 women. The study group was composed of 31 post-operative patients (26 post-cardiac surgery), 11 patients in septic shock from various medical conditions, 2 patients with severe gastrointestinal bleeding and 1 patient in congestive heart failure. Demographic and hemodynamic parameters for the group are listed in Table 1.

The mean SO_2 _and lactate concentrations for RA and PA blood samples are shown in Table 2. S_ra_O_2 _was greater than S_pa_O_2 _(p < 0.001), with ΔSO_2 _= 5.2 ± 4.8%. [Lac]_ra _was greater than [Lac]_pa _(p < 0.001), with Δ[Lac] = 0.2 ± 0.2 mmol.l^-1^.

**Figure 1:**
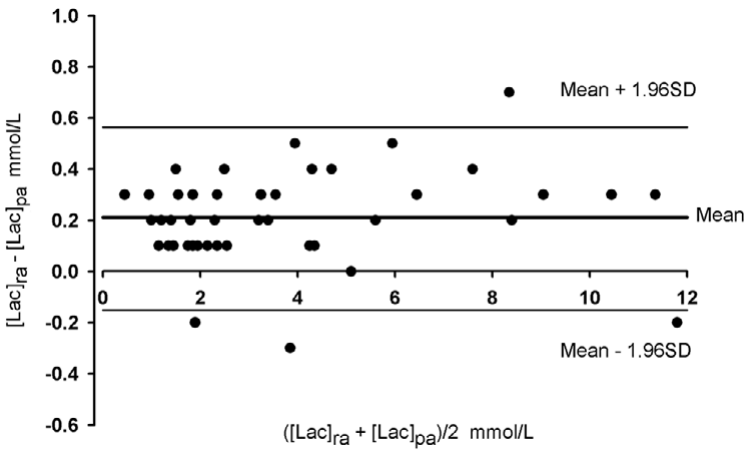
Bland-Altman plot comparing [Lac]ra and [Lac]pa. Bias 0.21 mmol.L-1 with a 95% confidence interval for the population of -0.15 to 0.56 mmol.L-1.

Shown in Fig. 1 is a Bland-Altman plot comparing [Lac]_ra _and [Lac]_pa_. There was a bias towards greater [Lac]_ra _of 0.2 mmol.l^-1 ^(p < 0.001) with a 95% confidence interval for the population of -0.15 to 0.56 mmol.l^-1^. There was no discernable relationship between [Lac]_ra _and Δ[Lac] (r^2 ^= 0.03; p = 0.33), indicating that Δ[Lac] was not a concentration dependent phenomenon. Moreover, we found no significant relationships between [Lac]_ra _and S_ra_O_2 _or between [Lac]_pa _and S_pa_O_2_.

**Figure 2:**
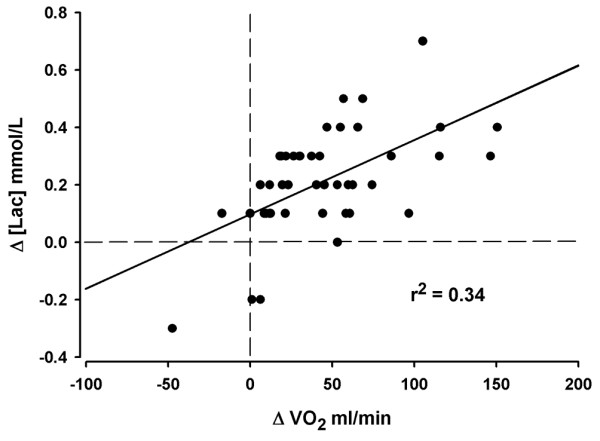
Linear correlation of Δ[Lac] to ΔVO2. The latter represents the difference in VO2 calculated using either SraO2 or SpaO2 in place of mixed venous SO2 in the Fick's Equation (Δ[Lac] mmol.L-1 = 0.0026 ΔVO2 ml.min-1 + 0.0975; r2 = 0.34; p < 0.0001). Standard error of the estimate 0.15 mmol.L-1.

There was a significant relationship between Δ[Lac] and ΔVO_2 _(Δ[Lac] mmol.l^-1 ^= 0.0026 ΔVO_2 _ml.min^-1 ^+ 0.0975; r^2 ^= 0.34; p < 0.0001) with a standard error of the estimate of 0.15 mmol.l^-1 ^(Fig. 2). There were no significant correlations between Δ[Lac] and cardiac index, DP, LVSWI, DO_2_, VO_2 _or ERO_2_.

## Discussion

We detected a decreasing Δ[Lac] when comparing paired blood samples drawn from the proximal and distal ports of PACs. We also noted Δ[Lac] correlated with ΔVO_2_. To our knowledge, these novel findings have not been reported elsewhere.

Only one other study in the literature has compared central venous [Lac] to [Lac]_pa_. This study found no differences in [Lac], although it was biased by the use of multiple blood samples (n = 50) drawn from 12 critically ill patients [12]. Our study used only one comparison per subject, which perhaps may explain the difference in results.

We used a standard clinical laboratory instrument to measure [Lac] having a 95% precision of ± 0.1 mmol.l^-1^. Even assuming a worst case scenario of a systematic instrument bias of -0.1 mmol.l^-1^, the difference in [Lac] between RA and PA would have remained statistically significant.

The declining [Lac] gradient from RA to PA is likely the result of mixing RA blood with blood of lower [Lac] emanating from the coronary venous system. Lactate oxidation accounts for 10% to 20% of total myocardial aerobic energy production [13], a proportion that increases substantially in sepsis [14]. As a result of myocardial lactate extraction, coronary venous [Lac] is substantially lower than arterial [Lac] and is the lowest of all venous effluents [15]. The dilution of RA blood by coronary venous blood of lower [Lac] is a plausible explanation for the small but detectable difference in [Lac] from RA to PA.

Since RA blood is the mixture of superior vena cava and inferior vena cava (IVC) blood, the possibility exists that these blood streams had not thoroughly mixed at the proximal PAC sampling port. In this case, one could expect further mixing to occur between IVC and RA blood while flowing into the pulmonary artery. Our results do not support this hypothesis. Direct measurements in humans show that IVC blood has the highest [Lac] of any major vein [9] and further mixing of RA with IVC blood would have produced higher, not lower, [Lac]_pa_. A factual resolution of this question can only be achieved by direct measurement of [Lac] from IVC to PA.

Only three individuals in our group had [Lac]_ra _< [Lac]_pa_. These patients had no distinguishing features to help us differentiate them from others in the group. It is possible that accidental mislabeling of the samples may have accounted for a negative Δ[Lac] but we think it unlikely, given the care taken with the labeling and measuring of the samples. Another possibility is that these individuals experienced myocardial ischemia, a condition associated with an upsurge in glucose metabolism and net lactate release by the heart [17-19]. Myocardial lactate release, as opposed to the normal state of myocardial uptake, would have resulted in [Lac]_ra _< [Lac]_pa_.

Others have noted a linear relationship between myocardial O_2 _consumption (MVO_2_) and myocardial lactate uptake, reflecting the O_2 _cost of lactate utilization by the heart [14]. We did not measure MVO_2 _directly but calculated ΔVO_2_, a parameter denoting the difference in systemic VO_2 _prior to and immediately after entry of myocardial effluent blood into the venous circulation. As such, ΔVO_2 _bears a direct relationship to MVO_2_. We noted a linear relationship between ΔVO_2 _and Δ[Lac] (Fig. 2) similar to that described between MVO_2 _and myocardial lactate uptake. This finding suggests that Δ[Lac] also may be related, in a yet to be established fashion, to MVO_2_.

## Conclusion

We found decreases in [Lac] from RA to PA in this sample of critically ill individuals. We conclude that parallel decreases in SO_2 _and [Lac] from RA to PA support the hypothesis that these gradients are produced by mixing RA with coronary venous blood of lower SO_2 _and [Lac]. The present study is a preliminary observation of this phenomenon and further work is needed to define the physiological and clinical significance of Δ[Lac].

## Key messages

• 	Oxygen and lactate concentrations are lower in PA blood than in RA blood.

• 	The oxygen and lactate concentration gradients from RA to PA are likely the result of mixing atrial with coronary venous blood.

• 	The possibility exists that these concentration gradients may reflect changes in myocardial energy requirements.

## Abbreviations

CV = coronary venous; CVP = central venous pressure; DO_2 _= systemic O_2 _delivery; DP = double product; ERO_2 _= oxygen extraction ratio; [Hb] = hemoglobin concentration; HR = heart rate; IVC = inferior vena cava; Δ[Lac] = lactate concentration gradient from right atrium to pulmonary artery; [Lac] = blood lactate concentration; LVSWI = left ventricular stroke work index; MAP = mean arterial pressure; MPP = mean pulmonary pressure; MVO_2 _= myocardial O_2 _consumption; PA = pulmonary artery; PAC = pulmonary artery catheter; PAOP = pulmonary artery occlusion pressure; RA = right atrium; SO_2 _= O_2 _saturation; ΔSO_2 _= O_2 _saturation gradient from right atrium to pulmonary artery; SVRI = systemic vascular resistance index; VO_2 _= O_2 _consumption.

## Competing interests

The authors declare that they have no competing interests.

## Authors' contributions

GG conceived the study, participated in its design, performed statistical analysis and drafted the manuscript. LSC and HZ participated in the design of the study, collected data and helped to draft the manuscript. MGS and NMK conducted the study, collected data and helped to draft the manuscript. All authors read and approved the final manuscript.

## Figures and Tables

**Table 1:** Study population demographic and hemodynamic parameters

Patient parameters (n = 45)	Mean ± SD
Age (years)	57.6 ± 13.2
APACHE II score	13.8 ± 6.0
HR (bpm)	92.1 ± 16.5
MAP (mmHg)	81.8 ± 13.0
MPP (mmHg)	27.6 ± 9.9
PAOP (mmHg)	18.6 ± 7.0
CVP (mmHg)	15.0 ± 6.1
Cardiac output (ml.min^-1^)	6.1 ± 2.6
Cardiac Index (ml.min^-1^.m^-2^)	3.3 ± 1.5
LVSWI (g.m.m^-2^.beat)	39.7 ± 12.6
DP (mmHg.beat.min^-1^)	7694 ± 1944
SVRI (dynes.sec.m^-5^)	2002 ± 1316
Hemoglobin (g.dl^-1^)	10.8 ± 2.0

CVP, central venous pressure; DP, double product (HR × MAP); HR, heart rate; LVSWI, left ventricular stroke work index; MAP, mean arterial pressure; MPP, mean pulmonary pressure; PAOP, pulmonary artery occlusion pressure; SVRI, systemic vascular resistance index.

**Table 2:** O_2 _saturation and lactate concentration of paired RA and PA blood samples

	RA blood	PA blood	Gradient (Δ)
O_2 _saturation (%)	74.2 ± 9.1 (53.1, 94.3)	69.0 ± 10.4^a ^(47.3, 90.5)	5.2 ± 4.8 (-8.1, 14.9)
Lactate concentration (mmol.l^-1^)	3.9 ± 3.0 (0.6, 11.7)	3.7± 3.0^a ^(0.3, 11.9)	0.2 ± 0.2 (-0.3, 0.7)

^a^P < 0.001 when comparing atrial to mixed venous blood by paired t-test. Mean ± SD; range shown in parenthesis; n = 45. RA, right atrium; PA, pulmonary artery.
